# Rare germline variants in pancreatic cancer and multiple primary cancers: an autopsy study

**DOI:** 10.1097/CEJ.0000000000000787

**Published:** 2023-03-02

**Authors:** Hiroo Fujitani, Hidetaka Eguchi, Yuta Kochi, Tomio Arai, Masaaki Muramatsu, Yasushi Okazaki

**Affiliations:** a Department of Molecular Epidemiology, Medical Research Institute, Tokyo Medical and Dental University; b Diagnostics and Therapeutics of Intractable Diseases, Intractable Disease Research Center, Graduate School of Medicine, Juntendo University; c Department of Genomic Function and Diversity, Medical Research Institute, Tokyo Medical and Dental University; d Laboratory for Autoimmune Diseases, RIKEN Center for Integrative Medical Sciences, Yokohama, Kanagawa; e Department of Pathology, Tokyo Metropolitan Geriatric Hospital, Tokyo, Japan

**Keywords:** DNA mismatch repair, double-strand break repair, gene panel sequencing, multiple primary cancer, pancreatic cancer, rare germline variant, variant of uncertain significance

## Abstract

There is a lack of information on rare germline variants of pancreatic cancer-predisposing genes. Risk genes for multiple primary cancers may overlap with those for pancreatic cancer.

**Methods:**

A retrospective study of autopsy cases with a negative family history in the Japanese single nucleotide polymorphism for geriatric research database examined rare germline variants in the protein-coding regions of 61 genes. Targeted sequencing of these genes was performed and classified for pathogenicity using the American College of Medical Genetics and Genomics guidelines. Polyphen-2, SIFT and LoFtool algorithms were used to predict damage to protein function.

**Results:**

Of the 189 subjects used (90 cancer and 99 non-cancer controls), 72 patients had pancreatic cancer (23 had multiple primary cancers) and 18 had no pancreatic cancer in multiple primary cancers. *APC, BRCA2, BUB1B, ENG* and *MSH6* were associated with cancer predisposition, and pathogenic/likely pathogenic (P/LP) variants occurred in 6% [pancreatic cancer (4/72); all-cancer (5/90)] and 54% (49/90) carried only variants of uncertain significance (VUS) among cancer patients. Of these VUS, in pancreatic cancer patients, four DNA mismatch repair (MMR) genes (*MLH1, MSH2, MSH6* and *PMS2*), and POLQ in men were significantly associated (odds ratio = 3.83; *P* = 0.025; *P* = 0.027, respectively). The most abundant predictor of functionally damaging variants was *POLQ*.

**Conclusions:**

The frequency of P/LP variants in patients with sporadic pancreatic cancer suggests the need for genetic evaluation of individuals with no family history. VUS of MMR genes (*MLH1, MSH2, MSH6* and *PMS2*) and *POLQ* may be useful in predicting genetic trends in the potential risk of pancreatic cancer, especially in individuals lacking P/LP.

## Introduction

Most patients with pancreatic cancer are sporadic (Vietri *et al*., 2022) and the incidence of pancreatic cancer is estimated to increase further. The risk factors for pancreatic cancer include sex, smoking, alcohol consumption, diabetes and family history. Pancreatic cancer is refractory and has a low 5-year survival rate. The etiology of pancreatic cancer is not fully understood. The close agreement between the incidence and mortality rates indicates that only a small fraction of patients are detected in the early stages of efficient intervention. These features highlight the need for effective detection methods for high-risk populations (Amundadottir *et al*., 2004; Kleeff *et al*., 2016). However, little is known about germline variants that predispose to pancreatic cancer.

Recent genetic analyses suggest that more than 5–10% of patients with pancreatic cancer carry rare germline variants such as *ATM, BRCA1, BRCA2* and Lynch syndrome (LS)-related four DNA mismatch repair (MMR) genes (*MLH1, MSH2, MSH6* and *PMS2*) (Solomon *et al.*, 2012; Lucas *et al.*, 2014; Jóri *et al.*, 2015; Hu *et al*., 2018; Hsu *et al.*, 2021). GWAS studies have been reported in patients with pancreatic cancer (Low *et al*., 2010; Willis *et al*., 2014; Roberts *et al*., 2016; Nakatochi *et al*., 2018; Mizukami *et al*., 2020). However, information regarding germline genes for pancreatic cancer in Japanese individuals without a family history is lacking.

Here, we aimed to provide information on rare germline variants in the elderly with pancreatic cancer, multiple primary cancers and non-cancers, using 61 gene panel sequencing. Most cancers occur in the elderly, imposing great public health and socioeconomic burden (Fane and Weeraratna, 2020). This case-control study is based on a database of consecutive autopsies registered for a study of geriatric disease, a design that characterizes the risk of pancreatic cancer in elderly individuals with a negative family history. Furthermore, this study is unique in that it includes centenarians of the same age and sex in the case and control groups.

## Methods

### Patient selection and ethics committees

Subjects for the case-control study were collected from consecutive autopsy samples of elderly individuals in Japan, referred to as JG-SNP: Japanese single nucleotide polymorphism for geriatric research (Sawabe *et al*., 2004) for the study of geriatric diseases. Autopsy samples were obtained from patients with pancreatic cancer and multiple primary cancers and those without cancer who were enrolled at Tokyo Metropolitan Geriatric Hospital between 1995 and 2012 and had no relevant family history. The control group consisted of non-cancer patients whose sex, age, smoking and alcohol consumption matched the patients in the case group as closely as possible. Patient medical records were referenced to obtain clinical data. Written informed consent was obtained from a family member of each patient involved in this study for the collection and research. This study was approved by the ethics committees of Tokyo Medical and Dental University (approval No. 02016-011), Tokyo Metropolitan Geriatric Hospital (approval No. R16-55) and Juntendo University (approval No. M18-0240).

### DNA extraction, quantification and quality control

DNA was extracted from the kidneys of autopsy samples using a standard phenol/chloroform extraction method. The purity and concentration of the DNA extracted from all samples were measured using a NanoDrop 1000 spectrophotometer (Thermo Scientific, Wilmington, Delaware, USA) and a Qubit fluorometer (Life Technologies, Carlsbad, California, USA). All the samples fulfilled the minimum quality and purity requirements of 1 mg in less than a 130-ml volume, with an optical density OD260/280 of 1.7–1.9 and an OD260/230 of greater than 2. DNA fragmentation was assessed by agarose gel (2%) electrophoresis.

### Panel sequencing

The 61 selected genes have previously been reported to be associated or potentially associated with cancer and are used by healthcare providers at our medical institution to predict the risk of cancer. AmpliSeq Custom panel was designed with AmpliSeq 7.0.2 (Thermo Fisher Scientific, Waltham, Massachusetts, USA) that covers exons, exon-intron boundaries, 5′UTR and 3′UTR regions of *AKT1* (Stoll *et al.*, 2005; Bleeker *et al.*, 2008)*, APC* (Elkharwily and Gottlieb, 2008)*, ATM* (Solomon *et al*., 2017)*, AXIN2* (Chan *et al*., 2022)*, BARD1* (Jain *et al.*, 2022)*, BMPR1A* (Voorneveld *et al.*, 2013)*, BRCA1, BRCA2* (Lucas *et al.*, 2014)*, BRIP1* (Mathew, 2006; Voutsadakis, 2020)*, BUB1*(Wu *et al.*, 2021)*, BUB1B* (Roberts *et al*., 2016; Rozengurt *et al*., 2018)*, BUB3* (Xiao *et al.*, 2022)*, CDH1* (Greil *et al*., 2022)*, CDKN1B* (Schernthaner-Reiter *et al*., 2016; Zhu *et al*., 2021)*, CHEK2* (Chaffee *et al*., 2018)*, CNTN6* (Garcia *et al.*, 2020)*, ENG* (Kokaji *et al.*, 2018; Hutton *et al*., 2021)*, EPCAM* (Went *et al.*, 2004; Ishiwata *et al.*, 2018)*, EPHX1* (Ockenga *et al.*, 2009; Sun *et al*., 2020)*, FANCC* (Rogers *et al.*, 2004)*, FANCE* (Waisfisz *et al.*, 1999)*, FAN1* (Smith *et al*., 2016), *GALNT12* (Guda *et al.*, 2009; Evans *et al.*, 2018)*, GREM1* (Chen *et al*., 2013; Lan *et al*., 2022)*, LRP6* (Garg *et al*., 2017)*, MBD4* (Bellacosa, 2001)*, MCM9* (Liu *et al*., 2016)*, MYH11* (Marcucci *et al*., 2020)*, MLH1, MLH3, MSH2, MSH3, MSH6* (Flores-Rozas and Kolodner, 1998; Berends *et al.*, 2002; Kastrinos *et al*., 2009; Bonadona *et al*., 2011; Adam *et al*., 2016; Gambichler *et al*., 2021)*, MUTYH* (Thibodeau *et al.*, 2019; Fountzilas *et al.*, 2021)*, NFKBIZ* (Kakiuchi *et al*., 2020; Xu *et al*., 2021)*, NTHL1* (Dhooge *et al*., 2020)*, PIK3CA* (Sivaram *et al*., 2019; Keane and Afghani, 2021)*, PMS1* (Roesner *et al*., 2013)*, PMS2* (Møller *et al*., 2018)*, POLD1* (Laquière *et al*., 2019; He *et al*., 2021)*, POLE* (Guenther *et al*., 2018)*, POLQ* (Higgins *et al.*, 2010; Wood and Doublié, 2016)*, PTEN* (Ren *et al*., 2022)*, RAD52* (Nogueira *et al*., 2019; Emelyanova *et al*., 2022)*, RBL1* (Friend *et al*., 1986; Naert *et al.*, 2020; Flores and Goodrich, 2022)*, REV3L* (Arivazhagan *et al*., 2017)*, RNF43* (Cancer Genome Atlas Research Network, 2017)*, RPS20* (Valle et al., 2019)*, SCG5* (Xu *et al*., 2020)*, SDHB* (Rahn *et al.*, 2019)*, SDHD* (Laitman et al., 2020)*, SMAD4* (Voorneveld *et al*., 2013)*, SMAD9* (Ngeow *et al*., 2015)*, SMARCA4* (Medina *et al*., 2008; Liu *et al*., 2014; Botta *et al*., 2021)*, STK11*(Petersen *et al*., 2006), *TDRD3* (Liu *et al*., 2018)*, TGFBR2* (Melisi *et al.*, 2021)*, TP53* (Hsiue *et al.*, 2021)*, UIMC1* (Basu *et al*.,2020)*, XAF1* (Huang *et al*., 2010) and *XRCC4* (Naumann *et al*., 2017) genes, together with upstream regions of *GREM1, APC* and *MSH2* (Eguchi and Okazaki, 2019). We used an Ion Chef instrument with Ion 520 and 530 kits (Thermo Fisher Scientific) for emulsion PCR, bead enrichment, and chip loading onto the Ion 530 chip (Thermo Fisher Scientific). The loaded chips were sequenced on an Ion GeneStudio S5 Plus sequencer (Thermo Fisher Scientific). The data obtained were analyzed using the Ion Reporter server 5.10 (Thermo Fisher Scientific). The variants were visually inspected using the Integrative Genomics Viewer [(IGV) Broad Institute].

## Variant evaluation

### Population frequency of variant carriers

We screened missense, nonsense and frameshift variants from panel sequences using a minor allele frequency (MAF) of less than 0.005 (0.5%) as a threshold in the jMorp 38KJPN dataset (version: 30 June 2022; accessed 30 December 2022) for approximately 38 000 Japanese individuals from the Tohoku Medical Megabank (https://jmorp.megabank.tohoku.ac.jp/202206/variants).

### Pathogenicity annotation

In this study, we included rare germline variants of pathogenic (P), likely pathogenic (LP), and of uncertain significance (VUS). First, we classified rare variants using the ClinVar (https://www.ncbi.nlm.nih.gov/clinvar) database for primary interpretation of their clinical importance (accessed 30 December 2022). Next, all variants except benign and likely benign were classified according to American College of Medical Genetics and Genomics (ACMG) guidelines (Richards *et al*., 2015). Supporting materials were obtained from National Center for Biotechnology Information (https://www.ncbi.nlm.nih.gov), gnomAD (https://gnomad.broadinstitute.org), COSMIC (https://cancer.sanger.ac.uk/cosmic) databases and InterVar (https://wintervar.wglab.org) bioinformatics software. Disease mutation in the Human Gene Mutation Database (https://www.hgmd.cf.ac.uk/ac/index.php) was noted for informative purposes (reference data: professional version 2022.2). Supplementary Figure 1, Supplemental digital content 1, http://links.lww.com/EJCP/A380 shows the variant classification process.

### Predicting functional effects of missense variants

We classified the annotations predicting the effect of missense variants on protein function using the following three algorithms in silico (accessed 30 December 2022): PolyPhen-2 (Polymorphism Phenotyping v2.2.3; http://genetics.bwh.harvard.edu/pph2), SIFT (https://sift.bii.a-star.edu.sg) and LoFtool (https://github.com/konradjk/loftee).

Polyphen-2 used the HumDiv model, an evaluation model for rare genes identified by genome-wide association studies, and the annotation by the respective algorithms of SIFT and LoFtool is mainly based on the Ensembl Variant Effect Predictor (https://asia.ensembl.org/Homo_sapiens/Tools/VEP). The predictive annotations for these three algorithms were classified as possibly damaging (PD), benign (B) for Polyphen-2, deleterious (D), deleterious low confidence (DLC), tolerated low confidence (TLC), tolerated (T) for SIFT, and PD, B for LoFtool.

### Statistical analysis

We statistically compared the associations between patients with and without cancer. In addition, the patients were examined separately according to their sex. The association between rare germline variants and cancer risk was estimated using the odds ratio (OR), confidence interval (CI), and *P* value for Fisher’s exact test and calculated using the MedCalc (https://www.medcalc.org) software. Disease prevalence and gene counts were compared between groups using SPSS statistics software (version 20 IBM, USA) and confirmed using the parametric Student’s *t*-test. The significance threshold for all tests was set at *P* < 0.05.

## Results

Patient characteristics are summarized in Table 1. The mean age of the patients was 82 years and ± 8.09 standard deviation (cancer patients: 82 ± 8.003; controls: 82 ± 8.21). Of the 90 patients with cancer, 72 (80%) had pancreatic cancer, including 49 with pancreatic cancer only (PaC) and 23 with pancreatic cancer and multiple primary cancers (PaMC). Eighteen (20%) patients had no pancreatic cancer in multiple primary cancers (NPaMC). The control group comprised 99 patients.

**Table 1:** Patient characteristics and demographics

Variable	Overall	Cancer patient	Control
	*N* = 189	*N* = 90	*N* = 99
Sex			
Women	105	49 (47%)	56 (53%)
Men	84	41 (49%)	43 (51%)
Age at death			
Mean ± *SD*	82 ± 8.09	82 ± 8.003	82 ± 8.21
Distribution			
100–104	2	1	1
90–99	31	14	17
80–89	83	40	43
70–79	61	30	31
61–69	12	5	7
Number of cancer*			
≥3		27	
2		14	
1		49	
Cancer phenotype			
Pancreatic (PaC)		49	
Pancreatic with multiple primary cancers (PaMC)		23	
No pancreatic in multiple primary cancers (NPaMC)		18	

Control, non-cancer patient; *N*, number; *SD*, standard deviation.

.*Per patient. Cancers diagnosed as primary were included.

**Fig. 1.**
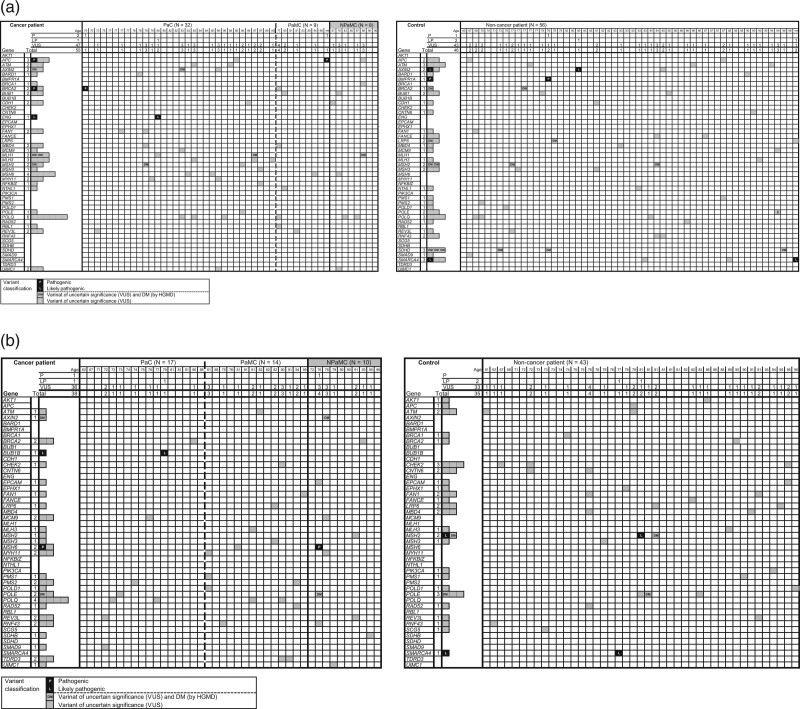
(a,b) Distribution of rare germline variants in cancer patients and controls. (a) Women. (b) Men. Rare germline variants were defined as those with minor allele frequencies <0.005 (0.5%). Case: cancer patient. Control: non-cancer patient. DM: disease mutation in HGMD database (professional version 2022.2). For pathogenic (P) or likely pathogenic (LP) variants with DM, these figures do not display DM (see Table 2 for details). In case one person carries more than one variant of the same gene, the number of variants is displayed in a single cell (Fig, 1a: *POLE* in control). HGMD, Human Gene Mutation Database; NPaMC: no pancreatic cancer in multiple primary cancers; PaC, pancreatic cancer only; PaMC, pancreatic cancer with multiple primary cancers.

We examined 61 cancer predisposition genes and identified 169 rare germline variants, including novel variants in 46 genes (Fig. 1a,b, Supplementary Table 1, Supplemental digital content 2, http://links.lww.com/EJCP/A381 and Supplementary Table 2, Supplemental digital content 3, http://links.lww.com/EJCP/A382).

**Table 2:** Rare germline pathogenic or likely pathogenic variants in cancer patients and controls

Patient	Sex	Age	Cancer phenotype	Gene	ACMG	Variant database		Variant	DNA change	Protein	db SNP	MAF
					classified	ClinVar	HGMD	effect		change		jMorp
Cancer	W	104	Pancreatic, duodenal, colorectal	*APC*	P	P	DM	Frameshift	c.4666dupA	p.Thr1556AsnfsX3	rs587783031	NR
	W	70	Pancreatic	*BRCA2*	P	P		Frameshift	c.3853_3854insG	p.Glu1285fs	rs2072490081	NR
	M	79	Pancreatic	*BUB1B*	LP	P/LP	DM	Missense	c.2441G>A	p.Arg814His	rs28989182	0.000039
	W	80	Pancreatic	*ENG*	LP	NR		Frameshift	c.728_729insCG	p.Pro244fs		NR
	M	76	Colorectal, esophageal	*MSH6*	P	P	DM	Nonsense	c.1444C>T	p.Arg482Ter	rs63750909	NR
Non-cancer	W	80		*AXIN2*	LP	NR		Frameshift	c.2063_2064insT	p.Thr689fs		NR
	W	78		*BMPR1A*	P	NR		Nonsense	c.441_442insT	p.Asp148Ter		NR
	M	81		*MSH2*	LP	CIP	DM	Missense	c.2197G>A	p.Ala733Thr	rs772662439	0.000517
	W	104		*SMARCA4*	LP	NR		Frameshift	c.153_154insC	p.Ala52fs		NR
	M	77		*SMARCA4*	LP	NR		Frameshift	c.153_154insC	p.Ala52fs		NR

Blank or NR indicates unknown or not reported.

Rare germline variants in the Japanese individuals registered in 38KJPN of the jMorp (Tohoku medical megabank organization [ToMMo]) genome database were examined with MAF <0.005 (0.5%). The final variant classification was performed following the guidelines of the American College of Medical Genetics (ACMG).

CIP, conflicting interpretations of pathogenicity; Control, non-cancer patient; DM, disease mutation in HGMD database (professional version 2022.2); dup, duplication; fs, frameshift; HGMD, Human Gene Mutation Database; ins, insertion. LP, likely pathogenic; M, man; MAF, minor allele frequency; P, pathogenic; VUS, variant of uncertain significance; W, woman.

### Pathogenic and likely pathogenic variants

Table 2 summarizes the rare germline P and LP variants. *APC* (P), *BRCA2* (P), *BUB1B* (LP), *ENG* (LP) and *MSH6* (P) were identified in patients with cancer.

The frequency of P/LP variants was 6% in each cancer group: pancreatic cancer (PaC + PaMC; 4/72), NPaMC (1/18) and all-cancer (5/90) groups. We identified *AXIN2* (LP), *BMPR1A* (P), *MSH2* (LP) and *SMARCA4* (LP; *n* = 2) in controls and the frequency of these variants (P/LP) was 5% (5/99). There was no statistically significant difference in the presence of pathogenic variants (P/LP) between cancer and non-cancer controls (*P* = 1.00).

### Variants of uncertain significance

We identified 159 rare germlines VUS. In cancer patients, 47 variants were identified in women and 36 variants were identified in men. In non-cancer patients, 43 variants were identified in women and 33 variants were identified in men. In the cancer group, 54% (49/90) of patients carried only VUS. The most frequent genes in patients with cancer were DNA polymerase theta (*POLQ; n* = 6) and *MSH6 (n* = 4) in women and *POLQ (n* = 4) in men (Fig. 1a,b).

We examined the association between VUS and the presence of cancer by using two statistical approaches in a case-control study. Table 3 summarizes the association between VUS and the presence or absence of cancer, using Fisher’s exact test.

**Table 3:** Association of rare germline variants of uncertain significance of four DNA mismatch repair genes (*MLH1, MSH2, MSH6* and *PMS2*) and *POLQ* in cancer patients and controls

Cancer type	Gene	Sex	Total (*N*)		Cancer patient (*N*)		Control (*N*)				Fisher’s exact
			Cancer	Control	aPresence	bAbsence	aPresence	bAbsence	OR	95% CI	*P* value
Pancreatic	*MSH6*	Combined	72	99	4	68	0	99	/	/	**0.029**
		Women	41	56	3	38	0	56	/	/	0.072
		Men	31	43	1	30	0	43	/	/	0.41
	*MLH1, MSH2, MSH6, PMS2*	Combined	72	99	10	62	4	95	3.83	1.15–12.75	**0.025**
		Women	41	56	7	34	3	53	3.63	0.87–15.03	0.09
		Men	31	43	3	28	1	42	4.5	0.44–45.47	0.3
	*POLQ*	Combined	72	99	8	64	3	96	4	1.02–15.64	0.054
		Women	41	56	4	37	3	53	1.9	0.40–9.04	0.45
		Men	31	43	4	27	0	43	/	/	**0.027**
All	*MSH6*	Combined	90	99	5	85	0	99	/	/	**0.023**
		Women	49	56	4	45	0	56	/	/	**0.044**
		Men	41	43	1	40	0	43	/	/	0.48
	*MLH1, MSH2, MSH6, PMS2*	Combined	90	99	13	77	4	95	4	1.25–12.79	**0.019**
		Women	49	56	9	40	3	53	3.97	1.01–15.63	0.06
		Men	41	43	4	37	1	42	4.54	0.48–42.45	0.19
	*POLQ*	Combined	90	99	10	80	3	96	4	1.06–15.03	**0.041**
		Women	49	56	6	43	3	53	2.46	0.58–10.43	0.29
		Men	41	43	4	37	0	43	/	/	0.052

The significance threshold was *P* < 0.05 and bold values indicate a significant difference.

Pancreatic: includes patients with multiple primary cancers.

All: all-cancer.

OR and CI were not calculated in the absence of patients and are indicated by (/). The most frequently identified VUS in cancer patients was *POLQ*, followed by *MSH6*.

CI, confidence interval; Control, non-cancer patient; MMR, DNA mismatch repair; *N*, number; OR, odds ratio; VUS, variant of uncertain significance.

a Presence: patient carrying variant.

b Absence: patient not carrying variant.

*MSH6*, four MMR genes (*MLH1, MSH2, MSH6* and *PMS2*), and *POLQ* were significantly associated with both pancreatic cancer and all-cancer groups. *MSH6* was significantly associated with pancreatic cancer (*P* = 0.029) and all-cancer groups (*P* = 0.023; women: *P* = 0.044). The ORs for MMR genes (*MLH1, MSH2, MSH6* and *PMS2*) were significantly higher in both the pancreatic cancer (OR = 3.83; *P* = 0.025) and all-cancer groups (OR = 4; *P* = 0.019). *POLQ* was significantly associated with pancreatic cancer in men (*P* = 0.027) and all-cancer groups (OR = 4; *P* = 0.041).

Table 4 summarizes the results of the Student’s *t*-tests. We examined the mean number of VUS for MMR genes (*MLH1, MSH2, MSH6* and *PMS2*).

**Table 4:** Mean number of rare germline variants of uncertain significance of four DNA mismatch repair genes (*MLH1, MSH2, MSH6* and *PMS2*) in cancer patients and controls

Cancer type	Sex	ACMG classified	Patient (*N*)		Mean number of variants		*t*-value	D*f*	*P* value
					per patient (*SD*)				
			Cancer	Control	Cancer	Control			
Pancreatic	Combined	VUS	10/72	4/99	0.13 (0.34)	0.04 (0.19)	2.15	104.1	**0.033**
	Women		7/41	3/56	0.17 (0.38)	0.05 (0.22)	1.75	60.56	0.08
	Men		3/31	1/43	0.09 (0.30)	0.02 (0.15)	1.25	41.15	0.21
All	Combined	VUS	13/90	4/99	0.14 (0.35)	0.04 (0.19)	2.46	136.85	**0.015**
	Women		9/49	3/56	0.18 (0.39)	0.05 (0.22)	2.04	74.82	**0.044**
	Men		4/41	1/43	0.09 (0.30)	0.02 (0.15)	1.41	58.69	0.16

The mean number of variants was compared between cancer and control groups using Student’s *t*-test. The significance threshold was *P* < 0.05 and bold values indicate a significant difference.

All, all-cancer; ACMG, American College of Medical Genetics and Genomics; Control, non-cancer patient; D*f*, degrees of freedom; MMR, DNA mismatch repair; *N*, number; Pancreatic, includes patients with multiple primary cancers; *SD*, standard devation; VUS, variant of uncertain significance.

The mean number of VUS for MMR genes (*MLH1, MSH2, MSH6* and *PMS2*) was significantly higher in the pancreatic cancer group (cancer patient, 0.13 vs. control, 0.04; *P* = 0.033) than that in the control group. In the all-cancer group, the mean number of VUS for MMR genes (*MLH1, MSH2, MSH6* and *PMS2*) was significantly higher (0.14 vs. 0.04; *P* = 0.015) than that in the control group. The mean number of VUS was significantly higher in the all-cancer group (0.18 vs. 0.05; *P* = 0.044) compared to the control group in women.

### MMR genes (*MLH1, MSH2, MSH6* and *PMS2*) in multiple primary cancers

One P variant and six VUS MMR genes (*MLH1, MSH2, MSH6* and *PMS2*) were identified in patients with multiple primary cancers (pancreatic, colorectal, gastric, bladder, cervical, esophageal, lung, skin and thyroid cancers). The details are presented in Supplementary Table 3, Supplemental digital content 4, http://links.lww.com/EJCP/A383.

### Predicting functional effects of missense variants

**Fig. 2.**
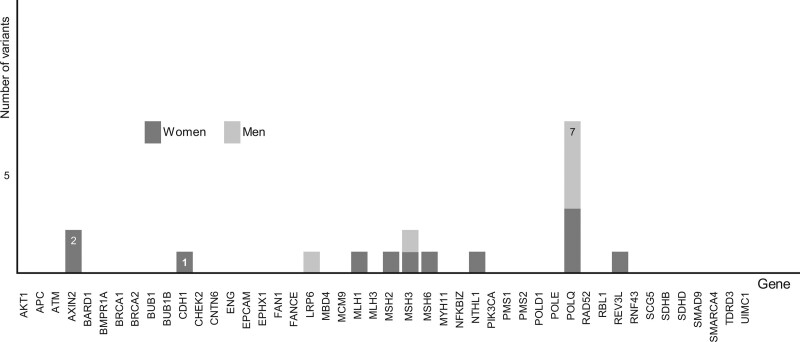
Predicted effects of variants of uncertain significance (VUS) in patients with pancreatic cancer. The missense variants were annotated as potentially damaging protein functions in Polyphen-2, SIFT, and LoFtool. VUS in pancreatic cancer patients with a total rating of possibly damaging/deleterious (PD/D) were listed. *POLQ* was the most frequently identified variant in this study. *CDH1* c.2638G>A (p.Glu880Lys) was also identified in one 67-year-old woman with hepatocellular carcinoma and colon cancer; not shown in this figure.

In silico analysis using Polyphen-2, SIFT and LoFtool was annotated in cancer patients and controls for predicted effects of missense variants on protein function (Supplementary Table 1, Supplemental digital content 2, http://links.lww.com/EJCP/A381 and Supplementary Table 2, Supplemental digital content 3, http://links.lww.com/EJCP/A382). All three algorithms listed VUS annotated as deleterious and possibly damaging in patients with pancreatic cancer (Fig. 2). *POLQ* was abundant (*n* = 7), and *AXIN2 (n* = 2), *MSH3 (n* = 2), *CDH1, LRP6, MLH1, MSH2, MSH6, NTHL1* and *LEV3L (n* = 1 each) were identified, and the same variant of *CDH1*, c.2638G>A (p.Glu880Lys), was also identified in one 67-year-old woman (NPaMC) with hepatocellular carcinoma and colon cancer.

During data collection, some information was unclear, such as the rs ID numbers for variants, but was scrutinized as closely as possible. Further updating of information on the data sets used in this study is needed.

## Discussion

We explored rare germline variants that encode proteins that predispose patients to sporadic pancreatic cancer. Panel sequencing of the 61 genes was performed using a case-control design in patients with a negative family history. Sequencing results revealed that 6% (4/72) of the patients, including one 104-year-old centenarian woman, with pancreatic cancer carried pathogenic cancer predisposition P/LP variants (Table 2). VUS of LS-related germline MMR genes and *POLQ* were significantly associated with pancreatic cancer. Interestingly, these results suggest that trends in cancer risk may differ in women and men, respectively (Tables 3 and 4).

Our report on the pathogenic frequency of rare germline variants provides evidence that reaffirms the importance of predicting pancreatic cancer risk using genetic screening in individuals without a family history. Previous studies have reported that the frequency of germline pathogenic variants in patients with familial pancreatic cancer ranges from approximately 5 to >10% (Bartsch *et al*., 2004; Petersen *et al*., 2006; Chaffee *et al*., 2018; Rainone *et al*., 2020), supporting the frequency of 6% observed in the present study. Pancreatic cancer-predisposing genes were also present in multiple cancer phenotypes, and similar results were observed for the following two cancer subsets: all-cancer (5/90) and NPaMC (1/18) groups. Despite current cost concerns, we speculate that comprehensive genetic evaluations, such as multigene panels, may be beneficial for early detection and therapeutic measures even for individuals with a negative family history.

We identified five genes associated with pancreatic cancer predisposition in patients with cancer. P/LP variants of *APC, BRCA2, BUB1B* and *ENG* were identified in patients with pancreatic cancer, and *MSH6* was identified in an NPaMC patient with both colorectal and esophageal cancers. No pathogenic P or LP variants were identified in any of these five genes in the control group (Table 2). In jMorp, a large Japanese database, *BUB1B* c.2441G>A (p.Arg814His) has been reported as a rare variant (MAF = 0.0039%); however, no records are available for *APC* c.4666dupA (p.Thr1556AsnfsX3), *BRCA2* c.3853_3854insG (p.Glu1285fs), *ENG* c.728_729insCG (p.Pro244fs) and *MSH6* c.1444C>T (p.Arg482Ter). The *ENG* c.728_729insCG variant is not listed in the ClinVar database (accessed 30 December 2022) and appears to be a novel variant. Meanwhile, the P/LP variants of *AXIN2, BMPR1A, MSH2* and *SMARCA4* were identified in 5% (5/99) of the subjects in the non-cancer control group, including one 104-year-old woman (Table 2), which did not significantly differ from the rate in the cancer group, and therefore the significance of risk is uncertain. These results also provide evidence that cancer risk prediction should be interpreted with caution and emphasize the complexity of understanding cancer mechanisms.

Germline variants can influence somatic variant patterns and genomic instability; ancestral populations with the same germline variants may exhibit different cancer phenotypes (Oak *et al*., 2020). One of our interesting findings is that *de novo APC* c.4666dupA (p.Thr1556AsnfsX3), which has been reported to cause an aggressive Gardner syndrome in a 2-year-old European boy (Kiessling *et al*., 2019), reproduced as a pancreatic, duodenal and colorectal cancer phenotype in a 104-year-old Japanese woman (Table 2). The effect of predisposition, such as family history, other genes, or environment, on the phenotypic differences between the two patients, i.e. the boy and the centenarian, remains unclear. Whether the pathogenic variant *APC* c.4666dupA varies significantly by sex, age and race in terms of disease phenotype, age of onset and prevalence require clarification.

Overall, the observed landscapes of rare germline variants were diverse and heterogeneous (Fig. 1a,b), suggesting that equivalent or complementary interactions between other susceptible genes and variants may influence cancer development. Of the 90 patients with cancer, 40% (36/90) carried no variants and 54% (49/90) carried only a potentially pathogenic variant, i.e. VUS.

We then examined whether VUS carriers of specific cancer predisposition genes are more likely to develop cancer. Our study evaluated the potential effects of VUS on the risk of developing cancer in a case-control study and confirmed statistical associations among the following genes: *MSH6*, four MMR genes (*MLH1, MSH2, MSH6* and *PMS2*) and *POLQ*. VUS for these genes was more abundant in patients with pancreatic cancer than in the non-cancer control group, and the same trend was observed in the all-cancer group (Tables 3 and 4). *MSH6* tends to delay cancer development compared to *MLH1* and *MSH2* (Kolodner *et al*., 1999; Berends *et al.*, 2002; Bonadona *et al*., 2011). Patients with multiple primary cancers (PaMC and NPaMC) with variants in *MLH1, MSH2, MSH6* and *PMS2* showed a trend consistent with various LS-related cancers (Geary *et al*., 2008; Kastrinos *et al*., 2009; Weissman *et al*., 2012; Win *et al*., 2012; Møller *et al*., 2018; Gambichler *et al*., 2021), although whether they had LS was unknown (Supplementary Table 3, Supplemental digital content 4, http://links.lww.com/EJCP/A383). In the results of the three algorithms for protein function prediction (Fig. 2, Supplementary Table 1, Supplemental digital content 2, http://links.lww.com/EJCP/A381 and Supplementary Table 2, Supplemental digital content 3, http://links.lww.com/EJCP/A382), the predicted impact of VUS on gene expression in patients with pancreatic cancer included predicted possibly damaging and deleterious variants in *MLH1, MSH2* and *MSH6*. Interestingly, *POLQ* were more abundant than those of other genes and some VUS might be loss-of-function variants in DNA double-strand break repair-related pathways. Genetic *POLQ* dysregulation plays an important role in genomic double-strand break repair and genome maintenance and is associated with the risk of pancreatic, breast, ovarian and other cancers, and overexpression is associated with poor prognosis (Higgins *et a*l., 2010; Wood and Doublié, 2016; Slater *et al*., 2021). Here, our results also revealed that the VUS of *MSH6* and *POLQ* suggested potentially different trends in association with cancer in women and men, respectively (Table 3). Our results may indicate that VUS in cancer patients may affect the potential accumulation of cancer risk, although there is no evidence of a causal relationship between VUS and cancer risk at this stage.

We could not examine microsatellite instability (MSI) as it relates to error repair in the germline; therefore, the association between MSI and the VUS of MMR genes is unknown. MMR gene-related cancers, including pancreatic cancer, are associated with MSI and MMR gene dysfunction, and the presence of MMR gene dysfunction or MSI-high is a known LS predictor and increases cancer risk (Laghi *et al*., 2012; Hause *et al*., 2016; Nojadeh *et al*., 2018). MSI status is also interesting because it predicts immunotherapy susceptibility such as immune checkpoint inhibitors (Baretti and Le, 2018; Latham *et al*., 2019; Ghidini *et al*., 2020). Previous studies that focused on sex differences in MSI in somatic MMR gene variants reported significant sex differences in esophageal and gastric cancers as well as an association among the differences in the incidence, prognosis, and treatment response of cancer (Li *et al*., 2018; Kohlruss *et al*., 2021). Further research on germline variants including MSI may facilitate the development of personalized therapies that focus on sex-based differences.

Information retrieved from the ClinVar database indicates no consensus on the involvement of VUS in cancer predisposition. The number of individual variant carriers of this study was limited and the small sample size was insufficient to establish the potential role of VUS in cancer. Nevertheless, the potential pathogenic risk of VUS is important for variant carriers. The clinical management of genetic diagnosis with risk prediction using VUS has several limitations. The current scrutiny of VUS pathogenicity has not kept pace with the number of VUS that have been detected. Indeed, many VUS are subsequently downgraded to benign variants whereas some are classified as pathogenic (Solomon *et al*., 2017; Macklin *et al*., 2018). In addition, a study also reported that the coexistence of VUS in MMR genes with predicted nonpathogenicity with other VUS of MMR genes might subtly increase cancer risk (Kantelinen *et al*., 2012). Hence, identification of VUS, which is very common in patients with cancer, and the collection of information on at-risk VUS by functional and epidemiological analysis is warranted. Findings of previous studies, as well as those of the current study, have suggested the importance of cancer risk prediction in individuals carrying rare germline VUS that lack the P/LP variants.

This study had several limitations. First, the rare germline variants identified in a population with a negative family history are likely *de novo* variants. However, no absolute evidence was found because no genetic information was available from the parents, families or relatives. Second, the effects of rare germline variants may be influenced by disease combination, smoking and drinking status, ethnicity, geographical region and database classification interpretation. Thus, the extent of variant reflection in the development of cancer remains unclear. Third, the presence of variants that were not included in the cohort and the uncertainty of the statistical power may stem from the small population size. Our results reflect only partial information on the individual patients in the study cohort. Prospective case-control studies including all age groups in various large populations of different ancestries are warranted to validate the current study findings.

In conclusion, our results provide important evidence for P/LP variants in 6% of elderly patients with pancreatic cancer with a negative family history. As for VUS, MMR genes (*MLH1, MSH2, MSH6* and *PMS2*) and *POLQ* may help predict potential trends in pancreatic cancer risk. *MSH6* and *POLQ* may have slightly different potential cancer-associated trends in women and men, respectively. Given the current limitations of pancreatic cancer risk prediction in the general population, algorithmic considerations for the VUS distribution of cancer-predisposing genes will be important in estimating trends in individual genetic risks in the future. Further studies are required to confirm these findings.

## Acknowledgements

The authors thank all the staff of the Department of Pathology Tokyo Metropolitan Geriatric Hospital Institute of Gerontology for preparing the samples. This study was supported in part by the Smoking Research Foundation (T.A.). The authors thank the following individuals for their contributions: Dr. Julien Legrand, Shizuoka University. Dr. Josvin K. Dr. Aye K.K Minn, Tohoku University. Dr. Moli G.

This study was partially funded by the Smoking Research Foundation (T.A.).

### Conflicts of Interest

There are no conflicts of interest.

## Supplementary Material








