# Supplementary material for: Rare germline variants in pancreatic cancer and multiple primary cancers: an autopsy study
Source: Eur J Cancer Prev. 2023 Mar 2;32(3):286–97. doi: 10.1097/CEJ.0000000000000787 (PMC10063194; doi:10.1097/CEJ.0000000000000787)
Supplement: Supplementary file 1 [file ejcp-32-286-s001.pdf]

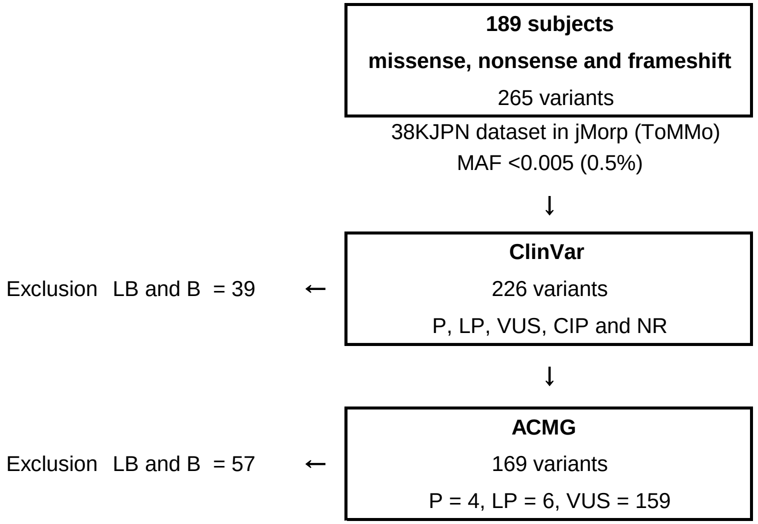

Supplementary Figure 1. Flow diagram of pathogenicity annotation of rare germline variants.
MAF: minor allele frequency. MAF is less than 0.005 (0.5%) in the Japanese individuals in the 38KJPN of the jMorp (Tohoku medical megabank organization [ToMMo]) genome database. P: pathogenic. LP: likely pathogenic. VUS: variant of uncertain significance. CIP: conflicting interpretation of pathogenicity. LB: likely benign. B: benign. NR: not reported. The final variant classification was performed following the guidelines of American College of Medical Genetics (ACMG).
