# Supplementary material for: Rare germline variants in pancreatic cancer and multiple primary cancers: an autopsy study
Source: Eur J Cancer Prev. 2023 Mar 2;32(3):286–97. doi: 10.1097/CEJ.0000000000000787 (PMC10063194; doi:10.1097/CEJ.0000000000000787)
Supplement: Supplementary file 2 [file ejcp-32-286-s002.pdf]

Supplementary Table 1. Overview of rare germline variants in elderly cancer patients and non-cancer controls: Women.

					ACMG	Variant database		Variant effect prediction			MAF jMorp	gnomAD					Total	Case (*N*)		Control
Gene	Variant effect	DNA change	Protein change	dbSNP	classified	ClinVar	HGMD	Polyphen-2	SIFT	LoFtool	JPN	EAS	SAS	EUR	LAM	AFR	(*N*)	Pancreatic	NPaMC	(*N*)
APC	missense	c.3875C>T	p.Thr1292Met	rs371113837	VUS	CIP	NR	B	D	PD	0.00226	0.0005765	0	0.0001176	0.000131	0.0001207	1	-	-	1
APC	frameshift	c.4666dupA	p.Thr1556AsnfsX3	rs587783031	P	P	DM	-	-	-	NR	NR	NR	NR	NR	NR	1	1	-	-
APC	missense	c.5201A>G	p.Lys1734Arg	rs1204161988	VUS	VUS	NR	PD	T	PD	0.000052	NR	NR	NR	NR	NR	1	1	-	-
APC	missense	c.6008A>G	p.Gln2003Arg	rs1580666656	VUS	VUS	NR	B	T	PD	NR	NR	NR	NR	NR	NR	1	-	1	-
APC	missense	c.8017A>G	p.Arg2673Gly	rs767286063	VUS	CIP	NR	PD	D	PD	0.001279	0.0003849	0	0	0	0	1	-	-	1
ATM	missense	c.125A>G	p.His42Arg	rs201773026	VUS	CIP	NR	B	T	B	0.001408	0.002499	0.0002068	0	0	0	1	1	-	-
ATM	missense	c.275A>C	p.Lys92Thr	rs200151849	VUS	CIP	NR	PD	T	B	0.000452	0.001153	0	0	0	0	1	-	-	1
ATM	missense	c.815A>G	p.Asn272Ser	-	VUS	VUS	NR	B	T	NR	0.000013	NR	NR	NR	NR	NR	1	-	-	1
ATM	nonframeshift	c.7740_7742delAAG	p.Arg2579del	-	VUS	NR	NR	-	-	-	NR	NR	NR	NR	NR	NR	1	-	-	1
ATM	missense	c.8246A>T	p.Lys2749Ile	rs779145081	VUS	CIP	NR	PD	D	B	0.001317	0.0003846	0	0	0	0	1	1	-	-
AXIN2	missense	c.1016G>A	p.Arg339His	rs2044042346	VUS	VUS	NR	PD	D	PD	NR	NR	NR	NR	NR	NR	1	1	-	-
AXIN2	missense	c.1177G>A	p.Glu393Lys	rs1555578459	VUS	VUS	NR	B	D	PD	0.000142	NR	NR	NR	NR	NR	1	-	-	1
AXIN2	frameshift	c.2063_2064insT	p.Thr689fs	-	LP	NR	NR	-	-	-	NR	NR	NR	NR	NR	NR	1	-	-	1
AXIN2	missense	c.2140C>T	p.Arg714Trp	rs148765149	VUS	CIP	DM	PD	D	PD	0.001498	0.002692	0	0	0	0	1	1	-	-
BARD1	missense	c.556A>G	p.Ser186Gly	rs16852741	VUS	CIP	NR	B	D	B	0.002236	0.0009619	0	0	0	0	1	1	-	-
BARD1	missense	c.2252G>A	p.Arg751Gln	rs587782246	VUS	VUS	NR	PD	D	B	NR	0	0	0.00004409	0	0	1	-	-	1
BMPR1A	nonsense	c.441_442insT	p.Asp148Ter	-	P	NR	NR	-	-	-	NR	NR	NR	NR	NR	NR	1	-	-	1
BRCA1	missense	c.626C>T	p.Pro209Leu	rs201596327	VUS	VUS	NR	B	T	PD	0.001201	0.0005769	0	0	0	0	1	-	1	-
BRCA2	frameshift	c.3853_3854insG	p.Glu1285fs	rs2072490081	P	P	NR	-	-	-	NR	NR	NR	NR	NR	NR	1	1	-	-
BRCA2	missense	c.5254C>A	p.His1752Asn	-	VUS	VUS	NR	B	T	NR	NR	NR	NR	NR	NR	NR	1	1	-	-
BRCA2	missense	c.9275A>G	p.Tyr3092Cys	rs80359195	VUS	CIP	DM	PD	D	PD	0.000039	0	0.0002076	0.00005882	0.000131	0.00002417	1	-	-	1
BUB1	missense	c.880G>A	p.Asp294Asn	rs561349655	VUS	NR	NR	B	D	B	0.000568	0.0007692	0	0.0000147	0	0	3	1	-	2
BUB1	missense	c.1600A>G	p.Asn534Asp	rs36109304	VUS	NR	NR	PD	D	B	0.002325	0.005202	0.0004144	0	0	0	1	-	1	-
CDH1	missense	c.2226_2227delGCinsCG	p.Pro743Ala	rs786203005	VUS	VUS	NR	B	T	PD	NR	NR	NR	NR	NR	NR	1	-		1
CDH1	missense	c.2638G>A	p.Glu880Lys	rs34507583	VUS	CIP	NR	PD	D	PD	0.003034	0.001153	0	0	0	0	2	1	1	-
CNTN6	missense	c.832T>G	p.Ser278Ala	rs775591980	VUS	NR	NR	B	T	B	0.002005	0	0.0002069	0	0	0	1	-	-	1
ENG	frameshift	c.728_729insCG	p.Pro244fs	-	LP	NR	NR	-	-	-	NR	NR	NR	NR	NR	NR	1	1	-	-
FAN1	missense	c.196A>G	p.Asn66Asp	-	VUS	NR	NR	B	T	NR	NR	NR	NR	NR	NR	NR	1	-	-	1
FAN1	missense	c.1063A>T	p.Ile355Phe	rs779723852	VUS	NR	NR	B	T	NR	0.000826	0.0003843	0	0	0	0	2	2	-	-
FANCE	missense	c.461C>T	p.Ser154Phe	rs1016962570	VUS	VUS	NR	B	T	B	0.000697	NR	NR	NR	NR	NR	1	-	-	1
FANCE	missense	c.1451C>G	p.Thr484Ser	rs1201037003	VUS	NR	NR	B	T	B	0.000413	NR	NR	NR	NR	NR	1	-	-	1
LRP6	missense	c.3926G>A	p.Gly1309Glu	rs1591868168	VUS	NR	DM	PD	D	PD	0.000039	NR	NR	NR	NR	NR	1	-	-	1
LRP6	missense	c.4568G>A	p.Arg1523Gln	rs200649441	VUS	NR	NR	PD	T	PD	0.003951	0.000966	0	0	0	0.00009697	1	-	-	1
MBD4	missense	c.125A>T	p.Glu42Val	rs758425374	VUS	NR	NR	B	DLC	PD	0.000142	NR	NR	NR	NR	NR	1	1	-	-
MBD4	missense	c.680A>G	p.Lys227Arg	rs199828331	VUS	NR	NR	B	T	PD	0.000956	0.0007683	0	0	0	0	1	1	-	-
MBD4	missense	c.1262A>G	p.Lys421Arg	rs147756381	VUS	NR	NR	PD	D	PD	NR	NR	NR	NR	NR	NR	1	-	-	1
MCM9	missense	c.1330G>C	p.Val444Leu	rs757364893	VUS	NR	NR	PD	D	NR	0.000529	0.0001928	0	0	0	0	1	1	-	-
MCM9	missense	c.2668T>C	p.Ser890Pro	-	VUS	NR	NR	NR	D	NR	NR	NR	NR	NR	NR	NR	1	-	-	1
MLH1	missense	c.1153C>T	p.Arg385Cys	rs63750760	VUS	CIP	DM	PD	D	PD	0.000968	0.000387	0	0.00005885	0	0.00002426	1	1	-	-
MLH1	missense	c.1572G>C	p.Met524Ile	rs587779953	VUS	VUS	NR	B	T	PD	0.001149	0.0005774	0	0	0	0	1	1	-	-
MLH1	missense	c.1744C>G	p.Leu582Val	rs63751713	VUS	VUS	DM	B	T	PD	0.001498	0.0003846	0	0	0	0	1	-	1	-
MLH3	missense	c.1153G>A	p.Asp385Asn	rs201389281	VUS	VUS	NR	B	T	PD	0.000065	0	0	0.0000588	0	0.00004823	1	1	-	-
MLH3	nonframeshift	c.1189_1191delATT_AAAT / A	p.Ile397del	rs777369430	VUS	CIP	NR	-	-	-	0.001356	0.000768	0	0	0	0	1	1	-	-
MLH3	missense	c.4015C>T	p.Leu1339Phe	rs201931206	VUS	CIP	NR	PD	D	PD	NR	0.0005783	0	0	0.00006547	0	1	-	-	1
MLH3	missense	c.4136G>A	p.Arg1379His	rs199974481	VUS	VUS	NR	PD	T	PD	0.000581	0.0001927	0	0.0000147	0	0.00002415	1	1	-	-
MSH2	missense	c.118G>A	p.Gly40Ser	rs63751260	VUS	CIP	DM	B	T	PD	0.003525	0.0001925	0	0	0	0	2	1	-	1
MSH2	missense	c.2064G>A	p.Met688Ile	rs63750790	VUS	CIP	NR	PD	D	PD	0.002337	0.0003849	0	0	0	0	1	1	-	-
MSH2	missense	c.2203A>G	p.Ile735Val	rs2229061	VUS	CIP	DM	PD	D	PD	0.00031	0.0001923	0	0.0000147	0	0.0003619	1	-	-	1
MSH3	missense	c.587C>T	p.Thr196Ile	rs774402842	VUS	VUS	NR	B	TLC	PD	NR	NR	NR	NR	NR	NR	1	-	-	1
MSH3	missense	c.833A>C	p.His278Pro	rs1749740358	VUS	VUS	NR	PD	D	PD	0.000362	NR	NR	NR	NR	NR	1	-	-	1
MSH3	missense	c.2125T>C	p.Phe709Leu	rs1805354	VUS	CIP	NR	PD	D	PD	0.003341	0.0003848	0	0	0	0	1	1	-	-
MSH6	missense	c.1223C>T	p.Pro408Leu	rs767404845	VUS	NR	NR	PD	D	PD	NR	NR	NR	NR	NR	NR	1	1	-	-
MSH6	missense	c.1364A>T	p.Asn455Ile	-	VUS	NR	NR	B	D	NR	NR	NR	NR	NR	NR	NR	1	1	-	-
MSH6	missense	c.1598A>C	p.Glu533Ala	-	VUS	NR	NR	B	T	NR	0.000039	NR	NR	NR	NR	NR	1	-	1	-
MSH6	missense	c.3202C>G	p.Arg1068Gly	rs63749843	VUS	VUS	NR	B	T	PD	0.000013	NR	NR	NR	NR	NR	1	1	-	-
MYH11	missense	c.28G>C	p.Asp10His	rs2043958315	VUS	NR	NR	PD	NR	NR	NR	NR	NR	NR	NR	NR	1	1	-	-
MYH11	missense	c.1889A>G	p.Asp630Gly	rs751232816	VUS	NR	NR	NR	D	PD	NR	NR	NR	NR	NR	NR	1	1	-	-
NFKBIZ	missense	c.295C>T	p.Pro99Ser	rs767220885	VUS	NR	NR	B	TLC	NR	NR	NR	NR	NR	NR	NR	1	1	-	-
NTHL1	missense	c.644C>T	p.Pro215Leu	rs755619109	VUS	NR	NR	PD	D	PD	NR	NR	NR	NR	NR	NR	2	1	-	1
PMS1	frameshift	c.1958delA	p.Lys653fs	rs764521118	VUS	NR	NR	-	-	-	0.000013	NR	NR	NR	NR	NR	1	-	-	1
PMS2	missense	c.472A>T	p.Ser158Cys	rs1554303942	VUS	VUS	NR	PD	D	PD	0.000232	NR	NR	NR	NR	NR	1	-	-	1
POLD1	missense	c.2678A>G	p.Asp893Gly	rs1249502531	VUS	VUS	NR	B	D	PD	0.001976	0.0001926	0	0	0	0	1	-	-	1
POLE	missense	c.2209A>G	p.Thr737Ala	rs779102091	VUS	VUS	NR	PD	D	B	0.000116	0.0003846	0	0.0000441	0	0.00002413	1	1	-	-
POLE	missense	c.3229C>G	p.Arg1077Gly	rs149777592	VUS	VUS	NR	PD	D	B	0.000129	0.0001925	0	0	0	0	1	-	-	1
POLE	frameshift	c.3232_3242delTACATCATCTC	p.Tyr1078fs	-	VUS	NR	NR	-	-	-	NR	NR	NR	NR	NR	NR	1	-	-	1
POLQ	missense	c.16C>G	p.Arg6Gly	rs55943551	VUS	NR	NR	B	TLC	PD	0.003215	0.005774	0.001244	0.0000147	0.0001309	0	1	-	-	1
POLQ	missense	c.17G>C	p.Arg6Pro	-	VUS	NR	NR	B	T	NR	0.000013	NR	NR	NR	NR	NR	1	-	1	-
POLQ	missense	c.196_197delAGinsTT	p.Arg66Leu	-	VUS	NR	NR	B	NR	NR	NR	NR	NR	NR	NR	NR	1	1	-	-
POLQ	missense	c.1151A>T	p.Gln384Leu	rs201034298	VUS	VUS	NR	B	T	PD	0.000813	0.001156	0	0.0000147	0.00006554	0	1	-	-	1
POLQ	missense	c.2450C>T	p.Thr817Ile	rs55923976	VUS	NR	NR	PD	D	PD	0.004649	0.0001924	0	0	0	0	1	1	-	-
POLQ	missense	c.6465G>T	p.Lys2155Asn	rs767522841	VUS	NR	NR	PD	D	PD	0.001989	0.0003852	0	0	0	0	1	1	-	-
POLQ	missense	c.6656G>A	p.Cys2219Tyr	rs202009405	VUS	VUS	NR	B	T	PD	0.000374	0.001538	0.000207	0	0	0	1	-	1	-
POLQ	missense	c.7318G>A	p.Val2440Ile	rs138273543	VUS	NR	NR	PD	D	PD	0.001898	0.001925	0	0	0	0	2	1	-	1
RAD52	missense	c.859A>C	p.Ser287Arg	rs772961926	VUS	NR	NR	B	T	B	0.000478	NR	NR	NR	NR	NR	1		-	1
RBL1	missense	c.2050G>A	p.Ala684Thr	rs374420710	VUS	NR	NR	B	T	PD	0.003422	0.00154	0	0	0	0	1	1	-	-
REV3L	missense	c.1721A>G	p.Lys574Arg	rs1780344182	VUS	NR	NR	PD	D	NR	0.000749	0.0001921	0	0	0	0	1	-	-	1
REV3L	missense	c.3830C>T	p.Pro1277Leu	rs1398400772	VUS	NR	NR	B	TLC	PD	0.001047	0.0001928	0	0	0	0	1	1	-	-
REV3L	missense	c.8591C>T	p.Pro2864Leu	rs199646165	VUS	NR	NR	PD	D	PD	0.004261	0.0005778	0	0	0	0	1	1	-	-
RNF43	missense	c.723C>G	p.Ser241Arg	-	VUS	NR	NR	B	NR	NR	NR	NR	NR	NR	NR	NR	1	-	-	1
RNF43	missense	c.809C>G	p.Pro270Arg	rs1474960950	VUS	NR	NR	B	D	PD	0.000013	0.0001923	0	0	0	0	1	-	-	1
SDHD	missense	c.331G>A	p.Val111Ile	rs201869798	VUS	VUS	DM	B	T	PD	0.003319	0.0003845	0	0	0	0	3	-	-	3
SMAD9	missense	c.88G>A	p.Glu30Lys	rs201033254	VUS	NR	NR	PD	D	PD	0.000452	0.0005785	0	0	0	0	1	-	-	1
SMARCA4	missense	c.44G>A	p.Gly15Asp	-	VUS	VUS	NR	PD	DLC	NR	NR	NR	NR	NR	NR	NR	1	-	-	1
SMARCA4	frameshift	c.153_154insC	p.Ala52fs	-	LP	NR	NR	-	-	-	NR	NR	NR	NR	NR	NR	1	-	-	1
SMARCA4	missense	c.964G>T	p.Ala322Ser	rs1060502089	VUS	VUS	NR	B	T	PD	NR	NR	NR	NR	NR	NR	1	-	-	1
UIMC1	missense	c.1574A>G	p.Asn525Ser	rs751506854	VUS	NR	NR	B	T	B	0.000013	0.0001925	0	0	0	0	1	-	1	-
UIMC1	missense	c.2012G>C	p.Arg671Pro	rs140185789	VUS	NR	NR	B	D	B	0.000504	0.000192	0	0	0	0	1	1	-	-

NR or (-) is not reported or unknown. *N* : number. ins: insertion. dup: duplication. del: deletion. fs: frameshift. P: pathogenic. LP: likely pathogenic. VUS: variant of uncertain significance. CIP: conflicting interpretations of pathogenicity. DM: disease mutation in HGMD database (professional version 2022.2).

The final variant classification was performed following the guidelines of American College of Medical Genetics (ACMG). We classified missense variants according to the potential for amino acid changes to protein function. Polyphen-2 used HumDiv, an evaluation model for rare genes identified by genome-wide association studies. SIFT and LoFtool annotations were done primarily by reference to Ensembl Variant Effect Predictor. Polyphen-2: possibly damaging (PD), benign (B). SIFT: deleterious (D), deleterious low confidence (DLC), tolerated low confidence (TLC), tolerated (T). LoFtool: possibly damaging (PD), benign (B).

MAF: minor allele frequency. Rare germline variants in the Japanese individuals registered in 38KJPN of the jMorp (Tohoku medical megabank organization [ToMMo]) genome database were examined with MAF <0.005 (0.5%).

JPN: Japanese, EAS: East Asian, SAS: South Asian, EUR: European (Non-Finnish), LAM: Latino/Admixed-American, AFR: African/African-American. Case: cancer patient. Control: non-cancer patient. Pancreatic: includes patients with multiple primary cancers. NPaMC: no pancreatic cancer in multiple primary cancers.
