# Supplementary material for: Rare germline variants in pancreatic cancer and multiple primary cancers: an autopsy study
Source: Eur J Cancer Prev. 2023 Mar 2;32(3):286–97. doi: 10.1097/CEJ.0000000000000787 (PMC10063194; doi:10.1097/CEJ.0000000000000787)
Supplement: Supplementary file 3 [file ejcp-32-286-s003.pdf]

Supplementary Table 2. Overview of rare germline variants in elderly cancer patients and non-cancer controls: Men.

					ACMG	Variant database		Variant effect prediction			MAF jMorp	MAF gnomAD					Total	Case (*N*)		Control
Gene	Variant effect	DNA change	Protein change	dbSNP	classified	ClinVar	HGMD	Polyphen-2	SIFT	LoFtool	JPN	EAS	SAS	EUR	LAM	AFR	(*N*)	Pancreatic	NPaMC	(*N*)
AKT1	missense	c.1373T>C	p.Met458Thr	rs587778018	VUS	VUS	NR	B	T	PD	0.000968	0.0007707	0	0	0	0.00009648	1	-	-	1
APC	missense	c.689G>A	p.Arg230His	rs587780545	VUS	CIP	NR	PD	D	PD	0.000271	0.0001924	0	0.0000147	0	0	1	-	-	1
ATM	missense	c.2942G>A	p.Arg981His	rs755896387	VUS	VUS	NR	B	T	B	0.000013	NR	NR	NR	NR	NR	1	-	-	1
ATM	missense	c.5108T>C	p.Phe1703Ser	rs772376652	VUS	VUS	NR	PD	D	B	NR	NR	NR	NR	NR	NR	1	-	-	1
ATM	missense	c.8246A>T	p.Lys2749Ile	rs779145081	VUS	CIP	NR	PD	D	B	0.001317	0.0003846	0	0	0	0	1	1	-	-
AXIN2	missense	c.2140C>T	p.Arg714Trp	rs148765149	VUS	CIP	DM	PD	D	PD	0.001498	0.002692	0	0	0	0	1	-	1	-
BRCA1	missense	c.154C>T	p.Leu52Phe	rs80357084	VUS	CIP	NR	PD	D	PD	0.003293	0.0003848	0	0	0	0	1	-	-	1
BRCA2	missense	c.1234C>A	p.Pro412Thr	rs1555281787	VUS	VUS	NR	B	T	PD	NR	NR	NR	NR	NR	NR	1	-	1	-
BRCA2	missense	c.5554G>A	p.Val1852Ile	rs80358777	VUS	CIP	NR	B	T	PD	0.000388	0.0001923	0	0.00002941	0	0	1	1	-	-
BRCA2	missense	c.10131A>C	p.Glu3377Asp	rs1064793835	VUS	VUS	NR	B	T	PD	0.000349	0	0	0	0	0	1	-	-	1
BUB1B	missense	c.2441G>A	p.Arg814His	rs28989182	LP	P/LP	DM	PD	D	B	0.000039	0	0	0.0000147	0	0	1	1	-	-
CHEK2	missense	c.1307T>G	p.Leu436Arg	rs878854914	VUS	VUS	NR	PD	D	PD	0.000103	NR	NR	NR	NR	NR	1	-	-	1
CHEK2	missense	c.755G>A	p.Ser252Asn	rs587781379	VUS	CIP	NR	B	T	PD	0.000762	0.0009608	0	0	0	0	1	1	-	-
CHEK2	missense	c.1513T>A	p.Ser505Thr	rs587781960	VUS	CIP	NR	B	T	PD	0.000736	0.0001925	0	0	0	0	2	-	-	2
CNTN6	missense	c.1069C>G	p.Arg357Gly	rs749841767	VUS	NR	NR	B	T	NR	0.000052	NR	NR	NR	NR	NR	1	-	-	1
CNTN6	frameshift	c.2683_2684insGTCA	p.Asn895fs	-	VUS	NR	NR	-	-	-	NR	NR	NR	NR	NR	NR	1	-	-	1
EPCAM	missense	c.298G>A	p.Asp100Asn	rs149274310	VUS	VUS	NR	B	D	B	0.001704	0.001156	0.0002074	0.00004411	0.00006558	0	1	-	-	1
EPCAM	missense	c.929G>C	p.Arg310Thr	rs1399510949	VUS	NR	NR	PD	D	B	0.00009	NR	NR	NR	NR	NR	1	-	1	-
EPHX1	missense	c.415C>T	p.His139Tyr	rs55784606	VUS	NR	NR	B	T	B	0.003022	0.004245	0	0	0.00006544	0	1	-	-	1
FAN1	missense	c.2761C>T	p.Arg921Cys	rs117225217	VUS	NR	NR	PD	D	NR	0.003848	0.005778	0	0.0000147	0	0	1	-	-	1
FAN1	missense	c.2906G>A	p.Arg969His	rs753068609	VUS	NR	NR	B	T	NR	0.001059	0	0	0	0	0.00002413	2	1	-	1
FANCE	missense	c.461C>T	p.Ser154Phe	rs1016962570	VUS	VUS	NR	B	T	B	0.000697	NR	NR	NR	NR	NR	1	-	-	1
LRP6	missense	c.2347C>T	p.Arg783Cys	rs1351098323	VUS	NR	NR	PD	T	PD	0.000052	0	0	0.0000147	0	0	1	-	-	1
LRP6	missense	c.3628G>T	p.Asp1210Tyr	rs372538850	VUS	NR	NR	PD	D	PD	0.000529	0.0003885	0	0	0	0	2	1	-	1
MBD4	missense	c.621T>A	p.Phe207Leu	rs578111412	VUS	NR	NR	B	T	NR	0.000052	NR	NR	NR	NR	NR	1	-	-	1
MBD4	missense	c.656G>T	p.Gly219Val	rs1460338276	VUS	NR	NR	PD	T	PD	0.001201	NR	NR	NR	NR	NR	1	-	-	1
MCM9	missense	c.2695C>G	p.Leu899Val	rs1047570524	VUS	NR	NR	B	TLC	NR	NR	NR	NR	NR	NR	NR	1	1	-	-
MCM9	missense	c.3419_3420delGAinsTT	p.Arg1140Ile	-	VUS	NR	NR	NR	D	NR	NR	NR	NR	NR	NR	NR	1	-	1	-
MLH3	missense	c.40C>T	p.Arg14Cys	rs1892532093	VUS	NR	NR	PD	D	PD	NR	NR	NR	NR	NR	NR	1	-	-	1
MLH3	missense	c.1560G>T	p.Glu520Asp	rs886050776	VUS	VUS	NR	B	T	PD	0.004198	0.0001922	0	0	0	0	1	1	-	-
MSH2	missense	c.2064G>A	p.Met688Ile	rs63750790	VUS	CIP	NR	PD	D	PD	0.002337	0.0003849	0	0	0	0	1	-	1	-
MSH2	missense	c.2197G>A	p.Ala733Thr	rs772662439	LP	CIP	DM	PD	D	PD	0.000517	0.0001925	0.0006219	0.0000294	0	0	1	-	-	1
MSH2	missense	c.2516A>G	p.His839Arg	rs63750027	VUS	CIP	DM	PD	T	PD	0.000168	0.001154	0	0	0	0	1	-	-	1
MSH3	missense	c.2005C>T	p.Arg669Trp	rs35045151	VUS	CIP	NR	PD	D	PD	0.000103	0	0	0.00001471	0.000131	0.002899	1	1	-	-
MSH3	missense	c.2125T>C	p.Phe709Leu	rs1805354	VUS	CIP	NR	PD	D	PD	0.003341	0.0003848	0	0	0	0	1	-	-	1
MSH6	nonsense	c.1444C>T	p.Arg482Ter	rs63750909	P	P	DM	NR	NR	NR	NR	0.0001926	0	0	0	0	1	-	1	-
MSH6	missense	c.1937A>G	p.Lys646Arg	rs201096652	VUS	CIP	NR	B	T	PD	0.000581	0.0001924	0	0	0	0	1	1	-	-
MYH11	missense	c.2645T>G	p.Leu882Arg	-	VUS	NR	NR	NR	D	NR	NR	NR	NR	NR	NR	NR	1	1	-	-
MYH11	missense	c.3457G>A	p.Glu1153Lys	-	VUS	NR	NR	NR	T	PD	NR	NR	NR	NR	NR	NR	1	-	1	-
PIK3CA	missense	c.1850G>A	p.Arg617Gln	rs3865687	VUS	VUS	NR	PD	T	PD	0.000854	0.0001928	0	0	0	0	1	-	-	1
PMS1	missense	c.1162A>C	p.Ile388Leu	rs774486487	VUS	NR	NR	B	T	PD	0.000646	0.0007683	0	0	0	0	1	1	-	-
PMS1	missense	c.2596T>G	p.Tyr866Asp	rs1373241137	VUS	NR	NR	PD	T	PD	NR	NR	NR	NR	NR	NR	1	-	-	1
PMS2	nonframeshift	c.1277_1279delTTC	p.Leu426del	-	VUS	NR	NR	-	-	-	NR	NR	NR	NR	NR	NR	1	1	-	-
PMS2	missense	c.1675G>A	p.Gly559Arg	rs751153838	VUS	VUS	NR	B	T	PD	0.000103	0	0	0	0.00006555	0.00007244	1	1	-	-
POLD1	missense	c.2678A>G	p.Asp893Gly	rs1249502531	VUS	VUS	NR	B	D	PD	0.001976	0.0001926	0	0	0	0	1	1	-	-
POLD1	missense	c.3256C>T	p.Arg1086Trp	rs963136799	VUS	VUS	NR	PD	D	PD	0.00235	0.00002943	0	0	0	0	1	-	-	1
POLE	missense	c.76A>G	p.Thr26Ala	rs182282150	VUS	VUS	NR	B	T	B	0.002557	0.0005778	0	0	0.0001309	0	1	-	-	1
POLE	missense	c.2974G>A	p.Ala992Thr	rs115193764	VUS	CIP	DM	B	T	B	0.004042	0.003846	0	0.0000294	0.00006545	0.00009656	2	-	1	1
POLE	missense	c.3983A>G	p.Asp1328Gly	-	VUS	NR	NR	PD	D	NR	NR	NR	NR	NR	NR	NR	1	-	-	1
POLE	missense	c.4649A>G	p.Lys1550Arg	rs5744947	VUS	VUS	NR	PD	T	B	0.00031	NR	NR	NR	NR	NR	1	1	-	-
POLQ	missense	c.1367G>A	p.Arg456His	rs375295723	VUS	VUS	NR	PD	D	PD	0.000065	0	0	0.0000147	0	0	1	1	-	-
POLQ	missense	c.2233C>T	p.Arg745Cys	rs373455498	VUS	NR	NR	PD	D	PD	0.000258	0	0	0.00005879	0.00006545	0.00002413	1	1	-	-
POLQ	missense	c.2450C>T	p.Thr817Ile	rs55923976	VUS	NR	NR	PD	D	PD	0.004649	0.0001924	0	0	0	0	1	1	-	-
POLQ	missense	c.2566C>T	p.Arg856Cys	rs200486636	VUS	NR	NR	PD	D	PD	0.003009	0.001153	0	0.0000147	0.00006543	0.00002412	1	1	-	-
RAD52	missense	c.164G>A	p.Arg55His	rs370272002	VUS	NR	NR	PD	D	B	0.004442	0.001733	0.0004137	0	0	0	1	-	-	1
RAD52	missense	c.742G>A	p.Ala248Thr	rs754974791	VUS	NR	NR	B	T	B	0.000374	0.001154	0.0002069	0	0	0	1	1	-	-
REV3L	missense	c.682G>A	p.Val228Ile	rs748613465	VUS	NR	NR	B	T	PD	0.000904	0.001731	0.0002068	0	0	0	1	1	-	-
REV3L	missense	c.1838C>T	p.Ser613Leu	rs55734490	VUS	NR	NR	PD	D	PD	0.001279	0.0003852	0	0	0	0	1	-	-	1
REV3L	missense	c.8591C>T	p.Pro2864Leu	rs199646165	VUS	NR	NR	PD	D	PD	0.004261	0.0005778	0	0	0	0	1	-	1	-
RNF43	missense	c.508G>A	p.Glu170Lys	rs757680292	VUS	VUS	NR	B	T	PD	0.002453	0.0003857	0	0	0	0	1	-	-	1
RNF43	missense	c.1309C>T	p.Arg437Trp	rs995358248	VUS	NR	NR	PD	D	PD	0.000039	0	0	0.0000147	0	0.00004825	1	-	1	-
RNF43	missense	c.1405G>C	p.Gly469Arg	rs775121749	VUS	NR	NR	PD	D	NR	0.000116	NR	NR	NR	NR	NR	1	1	-	-
SCG5	missense	c.119T>C	p.Ile40Thr	rs747076418	VUS	NR	NR	PD	DLC	PD	0.000039	NR	NR	NR	NR	NR	1	-	-	1
SDHB	missense	c.709C>T	p.Pro237Ser	rs186768244	VUS	CIP	NR	B	T	PD	0.000052	0.001156	0	0	0.00006548	0	1	-	1	-
SMAD9	missense	c.505C>T	p.Pro169Ser	rs199501242	VUS	NR	NR	B	T	PD	0.00124	0.0003874	0	0	0	0.00002414	1	1	-	-
SMARCA4	frameshift	c.153_154insC	p.Ala52fs	-	LP	NR	NR	-	-	-	NR	NR	NR	NR	NR	NR	1	-	-	1
TDRD3	missense	c.523A>G	p.Ile175Val	rs201745470	VUS	NR	NR	NR	T	PD	0.001035	0.001733	0	0	0	0	1	1	-	-
TDRD3	missense	c.2222G>A	p.Arg741Gln	rs184995173	VUS	NR	NR	NR	D	PD	0.004352	0.01058	0	0.0000735	0	0	1	1	-	-
UIMC1	missense	c.557C>G	p.Thr186Ser	rs758642310	VUS	NR	NR	B	T	B	0.001679	NR	NR	NR	NR	NR	1	-	1	-

NR or (-) is not reported or unknown. *N* : number. ins: insertion. dup: duplication. del: deletion. fs: frameshift. P: pathogenic. LP: likely pathogenic. VUS: variant of uncertain significance. CIP: conflicting interpretations of pathogenicity. DM: disease mutation in HGMD database (professional version 2022.2).

The final variant classification was performed following the guidelines of American College of Medical Genetics (ACMG). We classified missense variants according to the potential for amino acid changes to protein function. Polyphen-2 used HumDiv, an evaluation model for rare genes identified by genome-wide association studies. SIFT and LoFtool annotations were done primarily by reference to Ensembl Variant Effect Predictor. Polyphen-2: possibly damaging (PD), benign (B). SIFT: deleterious (D), deleterious low confidence (DLC), tolerated low confidence (TLC), tolerated (T). LoFtool: possibly damaging (PD), benign (B).

MAF: minor allele frequency. Rare germline variants in the Japanese individuals registered in 38KJPN of the jMorp (Tohoku medical megabank organization [ToMMo]) genome database were examined with MAF <0.005 (0.5%).

JPN: Japanese, EAS: East Asian, SAS: South Asian, EUR: European (Non-Finnish), LAM: Latino/Admixed-American, AFR: African/African-American. Case: cancer patient. Control: non-cancer patient. Pancreatic: includes patients with multiple primary cancers. NPaMC: no pancreatic cancer in multiple primary cancers.
