# Supplementary material for: Rare germline variants in pancreatic cancer and multiple primary cancers: an autopsy study
Source: Eur J Cancer Prev. 2023 Mar 2;32(3):286–97. doi: 10.1097/CEJ.0000000000000787 (PMC10063194; doi:10.1097/CEJ.0000000000000787)
Supplement: Supplementary file 4 [file ejcp-32-286-s004.pdf]

Supplementary Table 3. Rare germline variants of MMR genes (*MLH1, MSH2, MSH6,* and *PMS2*) in patients diagnosed with multiple primary cancers.

Sex	Age	Cancer phenotype	Gene	ACMG classified	Variant database		Variant effect	DNA change	Protein change	dbSNP	MAF
					ClinVar	HGMD					jMorp
W	88	Gastric, Thyroid	*MLH1*	VUS	VUS	DM	missense	c.1744C>G	p.Leu582Val	rs63751713	0.001498
M	91	Bladder, Gastric, Lung, Skin	*MSH2*	VUS	CIP		missense	c.2064G>A	p.Met688Ile	rs63750790	0.002337
M	76	Colorectal, Esophageal	*MSH6*	P	P	DM	nonsense	c.1444C>T	p.Arg482Ter	rs63750909	NR
W	76	Colorectal, Lung	*MSH6*	VUS	NR		missense	c.1598A>C	p.Glu533Ala		0.000039
M	81	Pancreatic, Thyroid	*MSH6*	VUS	CIP		missense	c.1937A>G	p.Lys646Arg	rs201096652	0.000581
W	94	Pancreatic, Colorectal, Cervical	*MSH6*	VUS	VUS		missense	c.3202C>G	p.Arg1068Gly	rs63749843	0.000013
M	94	Pancreatic, Colorectal, Gastric	*PMS2*	VUS	NR		nonframeshift	c.1277_1279delTTC	p.Leu426del		NR

Blank or NR indicates unknown or not reported. MMR: DNA mismatch repair. W: woman. M: man. del: deletion. The final variant classification was performed following the guidelines of American College of Medical Genetics (ACMG). P: pathogenic. VUS: variant of uncertain significance. CIP: conflicting interpretation of pathogenicity. DM: disease mutation in HGMD database (professional version 2022.2). MAF: minor allele frequency. Rare germline variants in the Japanese individuals registered in 38KJPN of the jMorp (Tohoku medical megabank organization [ToMMo]) genome database were examined with MAF<0.005 (0.5%).
